# P-1089. Community-based transmission networks of Staphylococcus aureus identified through hospital-based active surveillance and whole genome sequencing

**DOI:** 10.1093/ofid/ofaf695.1284

**Published:** 2026-01-11

**Authors:** Nawar Talukder, Courtney Takats, Gregory Putzel, Alice Tillman, Magdalena M Podkowik, Natalia M Arguelles, Anusha Srivastava, Julia Shenderovich, Alejandro Pironti, Bo Shopsin, Sarah E Hochman

**Affiliations:** NYU Langone, New Hyde Park, NY; NYU Langone Health, New York, New York; NYU Langone, New Hyde Park, NY; NYU Langone, New Hyde Park, NY; New York University, New York, New York; Washington University School of Medicine, St. Louis, Missouri; Case Western Reserve University School of Medicine, Cleveland, Ohio; NYU Langone Health, New York, New York; NYU Grossman School of Medicine, New York, New York; NYU Langone Health, New York, New York; NYU Langone Health, New York, New York

## Abstract

**Background:**

*Staphylococcus aureus* colonization precedes invasive infection. Hospitalized patients with *S. aureus* colonization can transmit to other patients, but community-based *S. aureus* transmissions are not well characterized. We used comprehensive, hospital-wide whole genome sequencing (WGS) of *S. aureus* isolates coupled with demographic data to characterize community-based *S. aureus* transmission clusters.

**Methods:**

8,567 *S. aureus* isolates from clinical and surveillance cultures collected between Oct 2022-Dec 2023 underwent WGS, with epidemiological and demographic data pulled from the electronic health record (EHR). Closely related isolates (< 20 single nucleotide polymorphisms) without in-hospital epidemiologic links underwent chart review, looking for community-based transmission clusters. Data was analyzed with Microsoft Excel.

**Results:**

The five largest clusters of closely related isolates lacking in-hospital epidemiologic links were all methicillin-resistant (MRSA). Cluster 1 included 23 patients with clonal complex 8/USA300, 83% were male with mean age 41 years, with high rates of substance use (71%), HIV (52%), and self-reported men who have sex with men (MSM, 35%). Cluster 5 (8 patients, clonal complex 30) had similar demographics as cluster 1 but with higher rates of substance use (87%), with half of patients found down prior to admission. All clinical cultures in clusters 1 and 5 were from skin and soft tissue infections (SSTI). Patients in clusters 2 and 3 (n=27 combined, both clonal complex 5) all resided in Brooklyn, NY, with mean age 67-75 years, 75% had recent nursing home or group home exposure and were more likely to have bacteremia and no SSTI. Patients in cluster 4 (n=9, clonal complex 8) were children with a mean age of 2.5 years, 2/3 of whom had MRSA colonization detected while in the neonatal intensive care unit;1/3 had SSTI and were from Orthodox Jewish communities in Brooklyn.

**Conclusion:**

WGS of *S. aureus* isolates obtained in the hospital identified large community transmission clusters, highlighting a need to integrate community risk factors with hospital-based infection prevention strategies. Future interventions targeting risk profiles can enhance efforts to prevent hospital-acquired *S. aureus* infections.

**Disclosures:**

All Authors: No reported disclosures

